# Development of biomarker signatures associated with anoikis to predict prognosis in patients with esophageal cancer: An observational study

**DOI:** 10.1097/MD.0000000000039745

**Published:** 2024-10-04

**Authors:** Yunwei Liang, Xin Yin, Yinhui Yao, Ying Wang

**Affiliations:** a Department of Oncology, the Affiliated Hospital of Chengde Medical University, Chengde, China; b Chengde Academy of Agriculture and Forestry, Institute of Medicinal Animals and Plants, Chengde, China; c Department of Pharmacy, the Affiliated Hospital of Chengde Medical University, Chengde, China.

**Keywords:** anoikis, drug therapy, esophageal cancer, prognosis, signatures

## Abstract

Anoikis, a form of programmed cell death linked to cancer, has garnered significant research attention. Esophageal cancer (ESCA) ranks among the most prevalent malignant tumors and represents a major global health concern. To ascertain whether anoikis-related genes (ARGs) can accurately predict ESCA prognosis, we evaluated the predictive value and molecular mechanisms of ARGs in ESCA and constructed an optimal model for prognostic prediction. Using the Cancer Genome Atlas (TCGA)-ESCA database, we identified ARGs with differences in ESCA. ARG signatures were generated using Cox regression. A predictive nomogram model was developed to forecast ARG signatures and patient outcomes in ESCA. Gene set enrichment analysis (GSEA) was employed to uncover potential biological pathways associated with ARG signatures. Estimation of stromal and immune cells in malignant tumor tissues using expression data (ESTIMATE) and cell-type identification by estimating relative subsets of RNA transcripts analyses were used to assess differences in the immune microenvironment of the ARG signature model. Based on ARGs, the patients with ESCA were divided into high and low groups, and the sensitivity of patients to drugs in the database of genomics of drug sensitivity in cancer was analyzed. Finally, the correlation between drug sensitivity and risk score was then evaluated based on the ARG signatures. Prognostic relevance was significantly linked to the ARG profiles of 5 genes: MYB binding protein 1a (MYBBP1A), plasminogen activator, urokinase (PLAU), budding uninhibited by benzimidazoles 3, HOX transcript antisense RNA, and euchromatic histone-lysine methyltransferase 2 (EHMT2). Using the risk score as an independent prognostic factor combined with clinicopathological features, the nomogram accurately predicted the overall survival (OS) of individual patients with ESCA. Gene ontology (GO) enrichment analysis indicated that the primary molecular roles included histone methyltransferase function, binding to C2H2 zinc finger domains, and histone-lysine N-methyltransferase activity. GSEA revealed that the high-risk cohort was connected to cytokine-cytokine receptor interaction, graft-versus-host disease, and hematopoietic cell lineage, whereas the low-risk cohort was related to arachidonic acid metabolism, drug metabolism via cytochrome P450 and fatty acid metabolism. Drug sensitivity tests showed that 16 drugs were positively correlated, and 3 drugs were negatively correlated with ARG characteristic scores. Our study developed 5 ARG signatures as biomarkers for patients with ESCA, providing an important reference for the individualized treatment of this disease.

## 1. Introduction

Esophageal cancer, with increasing morbidity, ranks seventh globally among new cases. Ranked eighth in prevalence, it stands as the sixth top cause of fatalities due to cancer.^[1]^ The annual morbidity and mortality rates of esophageal cancer are estimated at 6.3 and 5.6 per 100,000 people, respectively. The 5-year survival rate for esophageal cancer is quite low, between 10% and 30%, owing to variations in the frequency of risk factors and cancer subtypes.^[2]^ According to the GLOBOCAN project, if the current incidence rates hold steady, there will be 957,000 new esophageal cancer cases and 880,000 fatalities by the year 2040.^[3]^ Currently, white light endoscopy is considered the best method for screening esophageal cancer. Nevertheless, its steep price and intrusive characteristics result in a low acceptance rate, restricting its application.^[4]^ Today, surgery, radiotherapy, and chemotherapy are the main treatments for ESCA. Despite immunotherapy and targeted therapy’s clinical potential, there are still few drugs that are effective enough for targeted therapy to produce a satisfactory treatment effect.^[5]^ Due to its accuracy, noninvasiveness, and individualized drug therapy prediction, it is an essential tool for early diagnosis and treatment of esophageal cancer. Ongoing studies concentrate on new blood biomarkers for the early identification of cancer, with some already being used in medical settings.^[6]^ Nevertheless, conventional blood tumor markers like carcinoembryonic antigen and squamous cell carcinoma antigen lack adequate sensitivity and specificity, making them unsuitable for clinical use.^[7]^ Consequently, it is crucial to discover dependable biomarkers for forecasting the outcomes of esophageal cancer patients and to uncover the fundamental processes involved.

Anoikis, a form of programmed cell death induced by separation from the extracellular matrix, is crucial for stopping cells from proliferating and adhering to unsuitable surfaces, thus preventing the spread to other organs.^[8]^ Initially identified in epithelial cells, anoikis is involved in the physiological processes that ensure development and tissue homeostasis.^[9]^ When the anoikis mechanism fails, cells might persist in suspension or grow in abnormal locations where the extracellular matrix is different from the initial protein. Preventing anoikis is strongly linked to the spread and persistence of cancer cells.^[10–12]^ Research indicates that cancer advancement and the spread of metastases are linked to resistance to anoikis, specifically through anchor-independent growth and the epithelial-mesenchymal transition. The disruption of anoikis regulation is a key feature of cancer, aiding in the spread to remote organs.^[13–15]^ Additionally, the tumor microenvironment (TME) fosters anoikis resistance in neighboring cancer cells by modulating matrix stiffness, secreting survival-promoting soluble factors, increasing oxidative stress, inducing epithelial-mesenchymal transition and self-renewal properties, and leading to metabolic imbalances in cancer cells.^[8]^ These functions help prevent cancer cell death and sustain survival signals after detachment, thereby resisting anoikis and emerging as a promising target for antimetastatic treatments.^[8]^

Anoikis-related genes (ARGs) are crucial in the development and spread of various cancers such as gastric,^[16]^ endometrial,^[17]^ lung,^[18]^ and breast cancer.^[19]^ Research indicates that CPT1A-driven fatty acid oxidation is crucial for anoikis resistance, and reducing LPCAT1, an enzyme involved in cholesterol production, can suppress the unattached proliferation of esophageal squamous cell carcinoma (ESCC) cells.^[20,21]^ Another study showed that S-phase kinase associated protein 2 amplification and overexpression protect cancer cells from anoikis, thereby promoting metastasis in ESCC.^[22]^ F806 binds to site Arg610 of β1 integrin, thereby inhibiting β1 integrin activation. Furthermore, the formation of local adhesion is inhibited, and the adhesion of cells to the extracellular matrix is reduced, which eventually leads to anoikis, thus inhibiting the proliferation of ESCC cells and inducing their apoptosis.^[23]^

While anoikis has been linked to the outcomes of various cancers, no prognostic evaluation of esophageal cancer focusing on ARGs has been conducted. Consequently, utilizing the expression profiles and clinical details of ARGs from the esophageal cancer public database, this research evaluated and deliberated on the prognostic significance of ARG markers in ESCA, aiming to support personalized treatment strategies for esophageal cancer.

## 2. Materials and methods

### 2.1. Data processing

Gene expression profiles, somatic mutation data, copy-number variation data, and clinical details (such as sex, age, survival duration, survival status, and tumor stage) were sourced from The Cancer Genome Atlas database. A total of 183 esophageal cancer samples and 13 samples from healthy subjects were downloaded. The GSE53624 dataset, comprising 119 tumor samples with clinical data, was sourced from the gene expression omnibus repository.^[24]^ Using the R package “limma,” we identified the differentially expressed genes (DEGs) in TCGA-ESCA. The adjusted *p*-values were evaluated with the false discovery rate correction method, and DEGs were determined by setting the cutoff at |log2FC|>1 and an adjusted *P*-value of ≤ 0.05. ARGs were extracted from GeneCards (https://www. genecards.org). A total of 652 ARGs were identified and listed (Supplemental Digital Content, http://links.lww.com/MD/N605). The “VennDiagram” package in R was employed to display the intersecting genes between DEGs and ARGs in ESCA. Afterward, we examined the relationship of ARGs with somatic mutation frequency, genetic location, and copy-number variation of the ARGs. Since the research did not include experiments on cells, tissues, or animals, there were no ethical considerations necessary.

### 2.2. Development of ARG signature

Patients with ESCA were grouped into clusters using principal component analysis (PCA). The R software package, “ConsensusClusterPlus”^[25]^ was used to identify the unsupervised clusters of TCGA-ESCA datasets. To validate the clustering, the techniques PCA, T-distributed stochastic neighbor embedding, and uniform manifold approximation and projection were employed using the R packages “broom,” “Rtsne,” and “UMAP,” respectively. The R packages “Survival” and “‘survminer”’ were employed to examine Kaplan–Meier survival curves for various clusters, while “ComplexHeatmap” was utilized to display the gene expression patterns of these clusters.

Differentially expressed ARGs were collected based on their interaction with the DEGs. Using the R software package “SVA,” the 2 data sets (ESCA and GSE53624) are merged to avoid batch effects. To further identify prognostic genes associated with ARG, a univariate Cox regression analysis was conducted. Later, least absolute shrinkage and selection operator and multivariate regression analyses were employed to remove genes causing overfitting. After calculating the risk scores according to ARGs, all cases were classified according to their median risk scores. The method for calculating the risk factor involved the following formula: risk factor = (ARG_1_ expression × Coe*f*_1_) + (ARG_2_ expression × Coe*f*_2_) +…+(ARG_n_ expression × Coe*f*_n_), where Coe*f*_n_ represents the correlation coefficient of prognostic genes and ARG_n_ represents the expression of prognostic genes. Patients diagnosed with ESCA were categorized into groups based on high- and low-risk. The analysis utilized the R packages “survival,” “forest plot” and “glmnet,” maintaining a significance threshold of *P ≤ *.05.

### 2.3. GO and GSEA

Examination of the ESCA cohort indicated genetic variations between the high-risk and low-risk groups. A GO analysis was performed to identify the potential pathways related to these differential genes. The GO analysis encompassed 3 categories: molecular functions, biological processes, and cell composition. The “ClusteProfiler” R package^[26]^ was utilized to perform GSEA in order to investigate the distinctions between high-risk and low-risk groups. A *P*-value of 0.05 or less was considered statistically significant.

### 2.4. Establishment of an ARS-Based Nomogram

Kaplan–Meier analysis was employed to assess the OS across various clusters of high-risk and low-risk categories. Receiver operating characteristic analysis was employed to assess the accuracy and reliability of prognostic predictions. To assess if ARG characteristics were standalone indicators of ESCA prognosis, both univariate and multivariate regression analyses were conducted on the clinicopathological attributes and ARG signatures. A cumulative risk curve was utilized to compare the outcomes of high-risk and low-risk groups. A clinicopathological nomogram based on ARG was developed to estimate overall survival at 1, 3, and 5 years. Calibration graphs were employed to evaluate the predictive accuracy of the model and to ascertain its capability in nomogram identification.^[27]^

### 2.5. Exploration of immune microenvironment and immune cell infiltration

ESTIMATEScore^[28]^ was utilized to examine the variation in immune cell infiltration between groups at low and high risk. ESTIMATEScore is useful for assessing ImmuneScores, which indicate the abundance of immune cells, and stromal scores, which reflect the quantity of stromal cells. In all ESCA mRNA samples, expression ratio matrixes were obtained using the cell-type identification by estimating relative subsets of RNA transcripts function in the R software. Based on the ARGs median expression levels, ESCA samples were categorized into 2 groups, and the importance of immune cell variations between the high and low expression groups was assessed. A *P*-value of 0.05 or less was considered statistically significant.

### 2.6. Correlation between drug sensitivity and risk score

In order to explore the connection between high- and low-risk groups and their sensitivity to chemotherapy drugs, data on drug responses and targeting pathways were gathered from the genomics of drug sensitivity in cancer. The Spearman correlation analysis of drug information and risk score, the relationship between risk score and drug sensitivity, and the association with the signaling pathway targeted by these drugs were analyzed. The R package “pRRophetic” was utilized to determine the semi-maximum inhibitory concentration (IC50) based on ARG traits, demonstrating their predictive capacity.^[29]^ The IC50 value can be used to measure the apoptosis-inducing ability of a drug. Generally, the stronger the induction ability, the lower the IC50 value. The IC50 value provides information on the resistance of a cell to a drug.

### 2.7. Statistical Analysis

The R software (4.2.2) was utilized to conduct these analyses. Predictive elements were assessed through Cox regression analysis and the K-M analysis. We carried out survival analysis through K-M estimation and compared groups using the Wilcoxon rank-sum test. A *P*-value of 0.05 or less was considered statistically significant.

## 3. Results

### 3.1. Determination of Differentially Expressed ARGs

**Figure 1.**
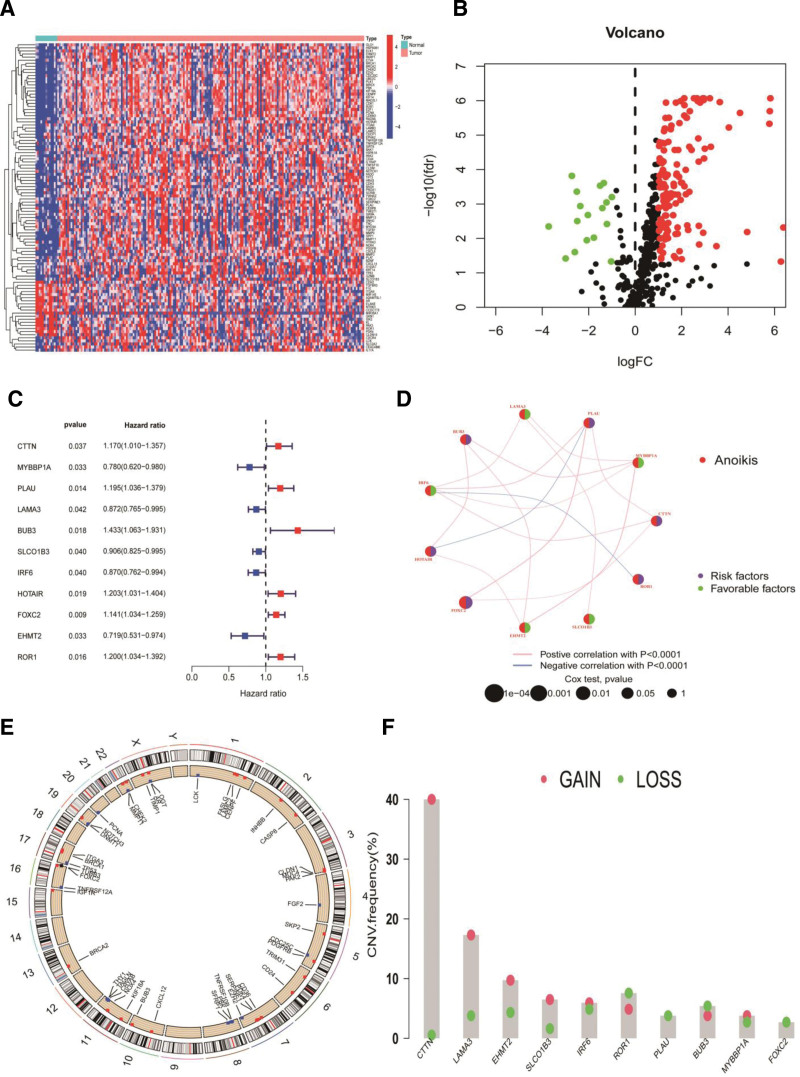
Characterization of differentially expressed ARGs. (A) Heatmap of DEGs in ESCA. (B) Volcano plot of DEGs in ESCA. (C) Forest plot of genes associated with prognosis in ESCA. (D) The network relationships of ARGs. (E) Rcircos of copy number variation. (F) The copy number variation frequency of genes associated with prognosis in ESCA. ARG = anoikis-related genes, DEG = differentially expressed genes, ESCA = esophageal cancer.

A total of 160 differentially expressed ARGs were obtained from the TCGA-ESCA database, including 18 downregulated and 142 upregulated genes (Figs. 1A, 1B). There were 18,687 genes after the 2 datasets (TCGA-ESCA and GSE53624) with esophageal cancer were merged without the batch effect. Univariate Cox analyses were performed to identify 160 ARGs in the merged datasets.

### 3.2. Construction of the ARG signatures

**Figure 2.**
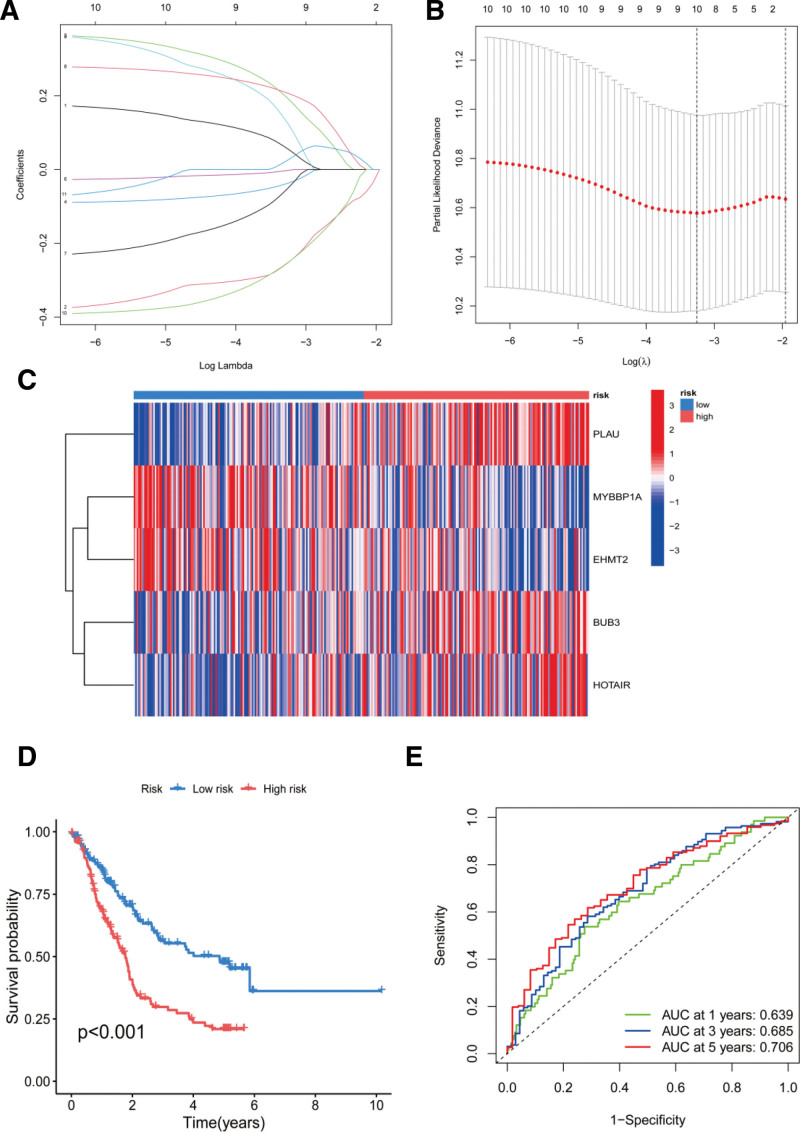
Identification of the ARS and construction ARGs signature. (A, B) LASSO regression analysis of ARGs in ECCA. (C) All patients were divided into high- and low-risk groups based on the risk score of each ESCA patient. (D) Survival differences between ESCA patients between high- and low-risk groups. (E) AUC of ROC in ESCA patients with 1, 3 and 5 years OS. ARG = anoikis-related genes, ECSA = esophageal carcinoma, OS = overall survival.

To develop an ARG signature, 11 prognostic ARGs were pinpointed through univariate analysis (Fig. 1C). The network relationships of the ARGs are shown in Figure 1D. Additionally, the copy-number variation frequencies of the 11 genes were calculated (Fig. 1E and 1F). Subsequently, the prognostic ARGs were overmatched using least absolute shrinkage and selection operator regression (Figs. 2A, 2B). Finally, 10 ARGs were screened. A multivariate Cox regression analysis was conducted to identify the 5-gene signatures. The calculation process is as follows: risk factor = (MYBBP1A × (−0.4940)) + (PLAU × 0.3281) + (BUB3 × 0.4077) + (HOTAIR × 0.3099) + (EHMT2 × (−0.3481)) (Figs. 2A, 2B).

### 3.3. Assessment and validation of ARG signatures

This equation was determined to calculate the risk score for every ESCA patient. Based on their scores, patients were categorized into groups of high- and low-risk (Fig. 2C). A significant difference in survival was observed between the 2 groups (Fig. 2D). The analysis of the ARG signature revealed that the AUC values of the receiver operating characteristic curves for 1-, 3-, and 5-year OS were 0.639, 0.685, and 0.706, respectively (Fig. 2E).

**Figure 3.**
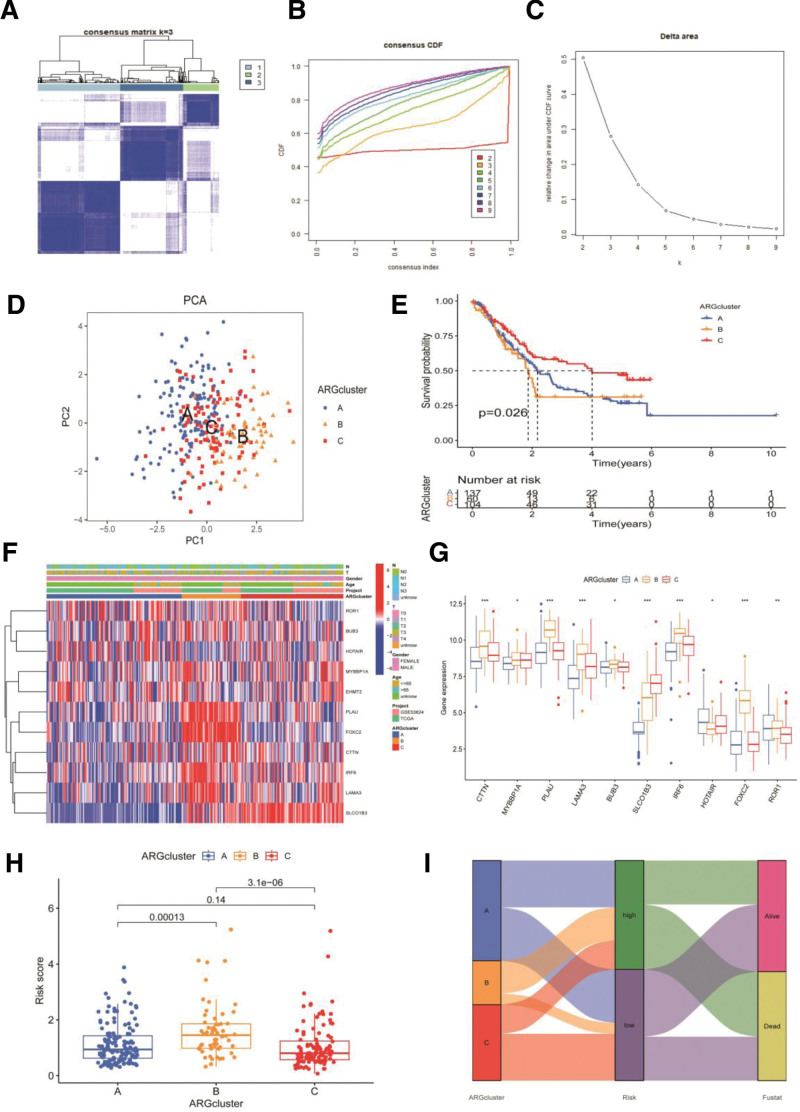
The PCA analysis was performed to obtain different clusters of ARGs in ESCA patients. (A–C) The patients were divided into clusters A, B, and C. (D) The PCA analysis of ESCA patients. (E) Survival differences between ESCA patients in 3 clusters. (F) Differential clinicopathological characteristics and OS in 3 clusters. (G) ARG cluster difference analysis. (H) Comparison of the ARGs among the 3 clusters. (i) Sankey diagram of 3 clusters. ARG = anoikis-related genes, ESCA = esophageal carcinoma, OS= overall survival, PCA = pSrincipal component analysis.

The PCA analysis was performed to obtain different clusters of ARGs in patients with ESCA. The results showed that k = 3 reached the clustering stability standard (Figs. 3A–3C). As a result, individuals with ESCA can be categorized into 3 clusters: clusters A, B, and C (Fig. 3D). Kaplan–Meier survival analysis of the 3 clusters found significant prognostic differences in ESCA patients (*P* < .05) (Fig. 3E). Over time, the survival rate of the patients showed a downward trend, with the prognosis of cluster C being better than that of clusters A and B. Figure 3F illustrates the distinct clinicopathological features and overall survival rates in ESCA clusters A, B, and C. The results of the ARG cluster difference analysis are shown in Figure 3G. The results indicated differences between clusters A and B, and clusters B and C (Figs. 3H, 3I).

### 3.4. Gene ontology and gene set enrichment analysis

**Figure 4.**
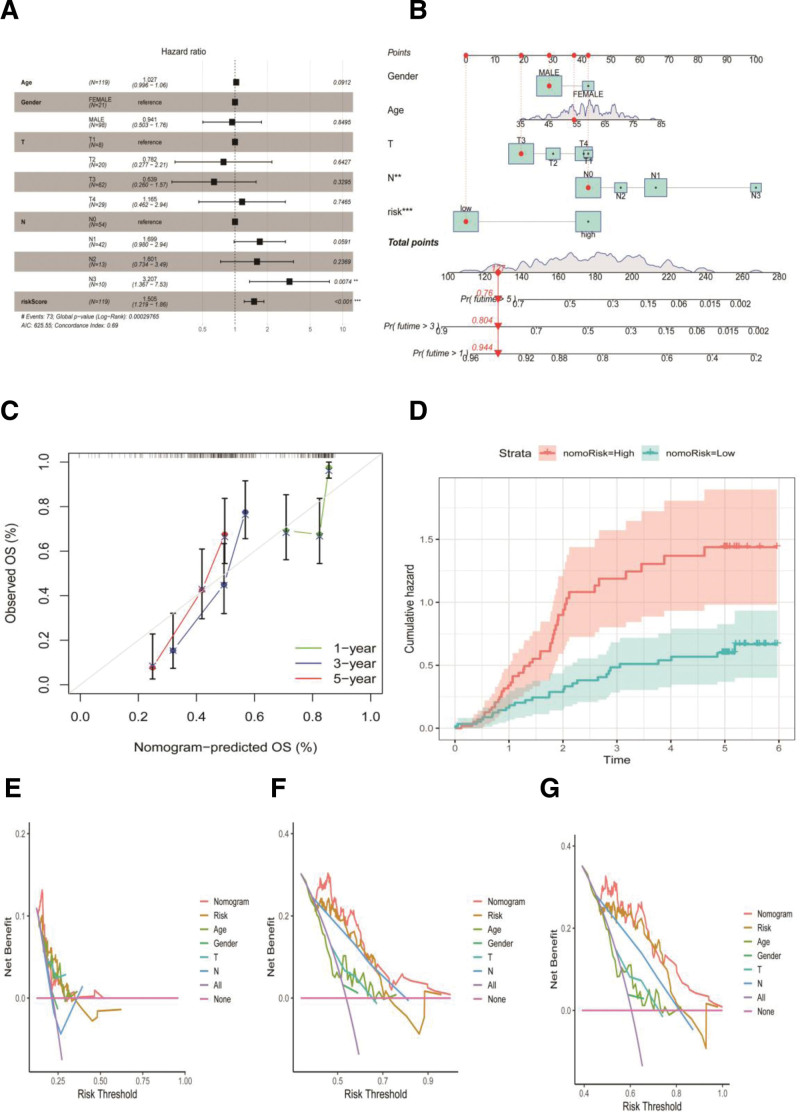
Construction of nomogram. (A) Independent prognostic analysis of ARGs signature and clinical characteristics. (B) The prediction model of nomogram. (C) Calibration curves of nomogram. (D) Cumulative hazard of nomogram. (E) ROC curve for 1 year. (F) ROC curve for 3 years. (G) ROC curve for 5 years. ARG = anoikis-related genes, ROC = receiver operating curve.

The “ClusterProfiler” package was utilized for GO enrichment analysis to pinpoint genes with differential expression between high-risk and low-risk groups (Fig. 4A). BPs included cellular responses to starvation, regulation of histone modifications, and responses to starvation. Cell composition included the nucleocytoplasmic transport, peptidase inhibitor, and serine-type endopeptidase complexes. Molecular functions included histone methyltransferase activity to H3-K9, C2H2 zinc finger domain binding, and histone-lysine N-methyltransferase activity.

The BPs involved in esophageal cancer with different levels of ARG expression were investigated using GSEA. When comparing the high-risk and low-risk groups, the high-risk group predominantly exhibited signaling pathways related to cytokine-cytokine receptor interaction, graft-versus-host disease, and hematopoietic cell lineage (Fig. 4B). The signaling pathways linked to low-risk included arachidonic acid metabolism, drug metabolism, cytochrome P450, and fatty acid metabolism (Fig. 4C).

### 3.5. Development of the nomogram

**Figure 5.**
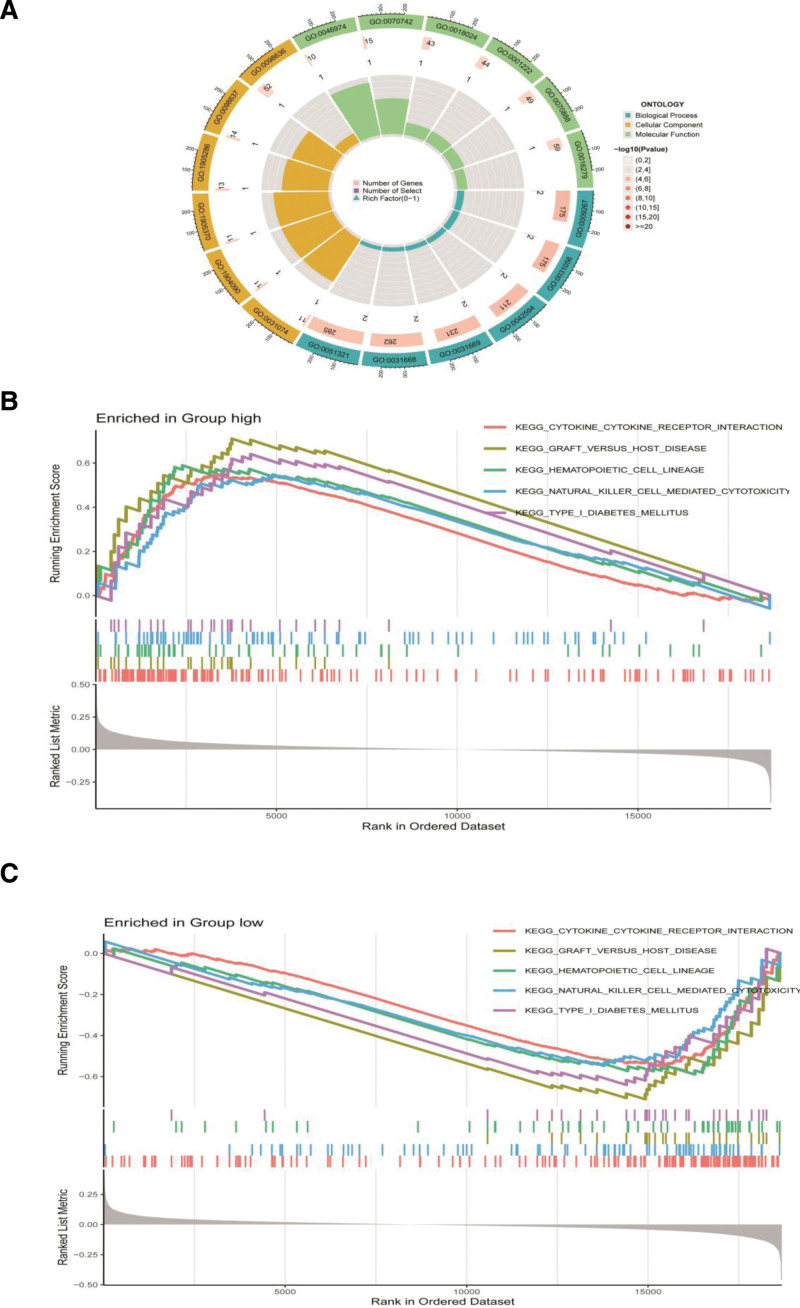
GSEA analysis of the ARS. (A): The ssGSEA analysis of ARG in 3 clusters. (B) GSEA analysis showed that only enriched in clusterB. ARS = anoikis-related signature, GSEA = gene set enrichment analysis.

To delve deeper into the predictive impact of ARG signatures, an independent analysis was conducted using ARG characteristics and clinical variables from the TCGA dataset. The findings indicated that variables like ARG signature scores and lymphatic node involvement were significant (*P* < .05).This evaluation revealed that the ARG signature risk score continued to be a significant prognostic factor, implying that the suggested anoikis-related signature (ARS) might operate independently of other clinical variables (Fig. 5A). To create a nomogram model tailored to clinical practice requirements, patient data including sex, age, TN stage, and ARG signatures for those with ESCA were collectively analyzed (Fig. 5B). Calibration curves (Fig. 5C) and cumulative hazards (Fig. 5B). Calibration curves (Fig. 5C) and cumulative hazards (Fig. 5D) show a strong correlation between the predicted and OS rates of patients with ESCA. Figures 5E–5G illustrate the decision curve analysis conducted for the overall survival rates at 1, 3, and 5 years.

### 3.6. Immune Activity Analysis of the ARS Model

**Figure 6.**
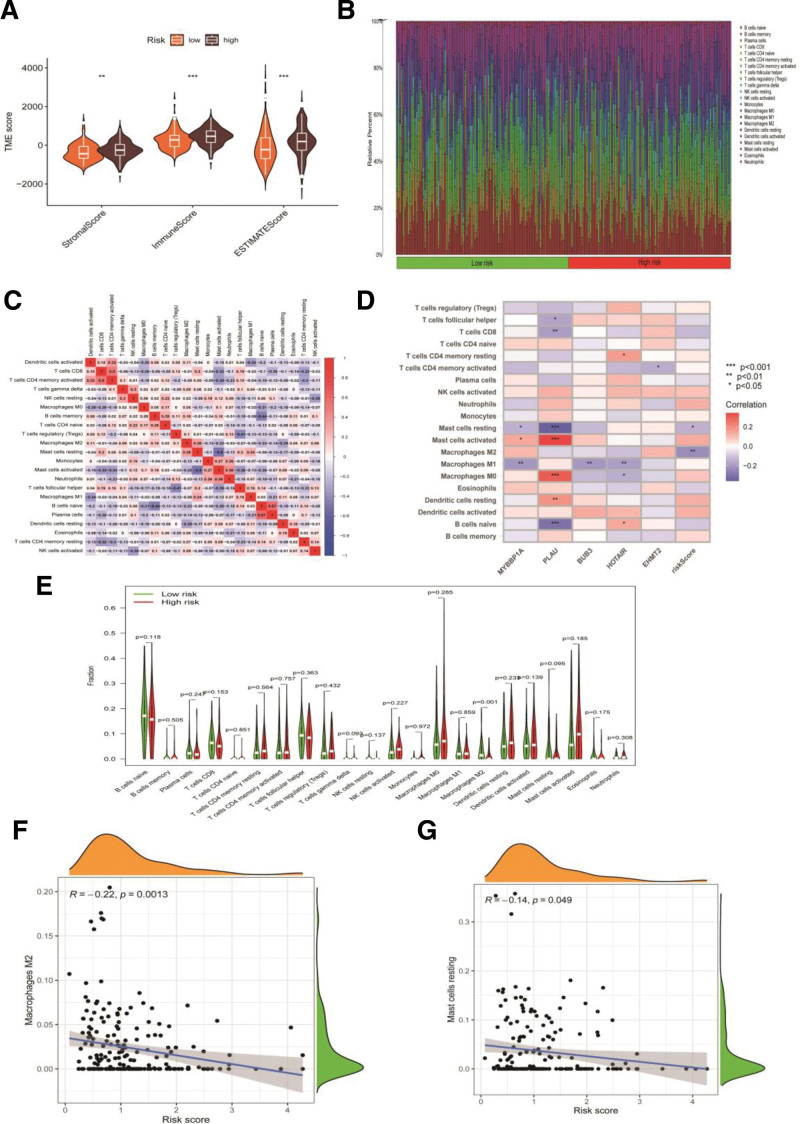
Immune activity analysis of the ARS in high- and low-risk groups. (A) Differences of immune microenvironment scores between high- and low-risk groups. (B) Heatmap of to assess immunotherapy response in high- and low-risk groups. (C) The results of immune cell correlation analysis. (D) Immune-related genes were associated with patients’ risk scores. (E) The differences of immune functions between the high-and low-risk groups. (F) Macrophages M2 is negatively correlated with the risk score of the patients. (G) Mast cells resting is negatively correlated with the risk score of the patients. ARS = anoikis-related signature.

A clear distinction exists between patients at low and high risk regarding TME, a crucial marker of tumor biological behavior in the ESCA group. Based on the ESTIMATE analysis, low-risk patients had lower ImuneScores, StromalScores, and ESTIMATEScores compared to that of high-risk patients (Fig. 6A).

The cell-type identification by estimating relative subsets of RNA transcripts algorithm was employed to examine the relationship between ARS and immune cell infiltration by analyzing the proportions of 22 different immune cell types (Figs. 6B–6E). Figure 6b presents a correlation heatmap illustrating the immunotherapy response results for both high-risk and low-risk groups. The results of the immune cell correlation analysis are presented in Fig. 6C. The analysis of immune correlations identified a link between genes related to immunity and the risk scores of patients (Fig. 6D). Figure 6E illustrates the variations in immune response between the high-risk and low-risk groups. A notable statistical disparity was observed between M2 macrophages and inactive mast cells, with their scores showing a negative correlation with patients’ risk factors (Figs. 6F and 6G).

### 3.7. Analyzing the relationship between drug response and risk assessment

**Figure 7.**
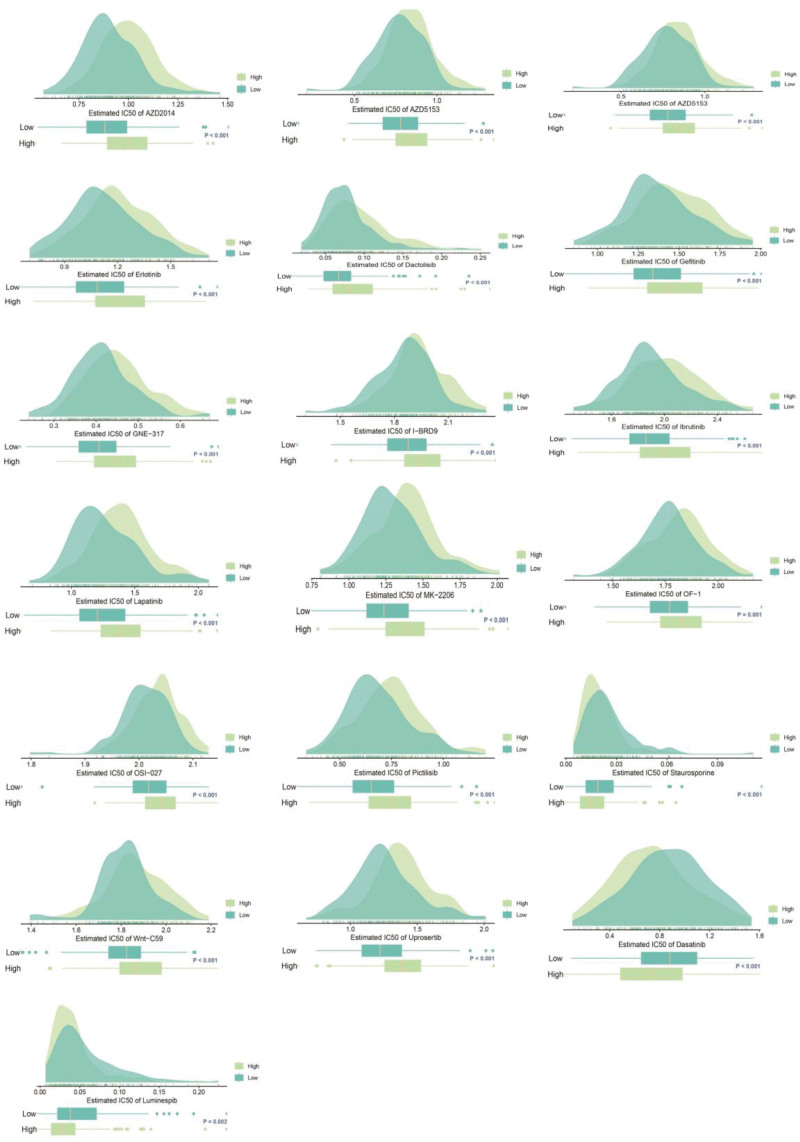
Drug sensitivity analysis in high- and low-risk groups.

Ultimately, the potential for drug resistance was examined between ESCA patients at high and low risk. In patients with ESCA, IC50 values were correlated with the IC50 scores of targeted and chemotherapy drugs (Fig. 7). The IC50 values of 16 drugs (Afuresertib, AZD2014, AZD5153, Dactolisib, Erlotinib, Gefitinib, GNE-317, I-BRD9, Ibrutinib, Lapatinib, MK-2206, OF-1, OSI-027, Pictilisib, Uprosertib and Wnt-C59) showed a significant positive correlation with patient risk scores, suggesting that these drugs may not be effective in patients with high-risk ESCA. Conversely, the IC50 values of 3 drugs (Staurosporine, Dasatinib, and Luminespib) exhibited a strong inverse relationship with patient risk scores, suggesting these medications could be beneficial for high-risk ESCA patients.

## 4. Discussion

ESCA is a frequently occurring cancer with a grim outlook, having a 5-year survival rate below 5%.^[30]^ Owing to the lack of reliable prognostic biomarkers ESCA is difficult to diagnose in its early stages. Consequently, it is crucial to discover dependable prognostic markers to improve the prediction of ESCA outcomes. Research indicates that anoikis, a distinct type of programmed cell death, influences the biological behavior of different cancers.^[8]^ Jin et al^[18]^ showed that glutamate dehydrogenase 1 levels increase after pleomorphic adenoma gene 1 dissociation, offering antianoikis and metastasis-promoting signals in lung cancer lacking LKB1. Zhang et al^[31]^ used proteomics to identify anoikis suppressors, revealing that low expression of IL1 receptor accessory protein in normal human tissues is a promising immunotherapeutic target in Ewing Sarcoma. circRNA generated from cell migration-inducing protein boosts defensive autophagy in prostate cancer cells, leading to increased resistance to anoikis.^[32]^ A different research demonstrated that the activation of catenin beta 1 transcription by nuclear myosin heavy chain 9 resulted in resistance to anoikis and promoted metastasis in gastric cancer cells, both in vitro and in vivo.^[33]^ Consequently, ARGs could serve as prognostic indicators and treatment targets for a range of cancers.

For our research, we obtained clinical data and gene expression profiles associated with ESCA from TCGA and gene expression omnibus databases and identified differentially expressed ARGs in ESCA using the TCGA-ESCA dataset. The patients were split into training and test groups, followed by univariate and multivariate Cox regression analyses, leading to the creation of a prognostic risk score based on 5 ARGs. The outlook varied greatly between the high- and low-risk groups. The model’s precision was confirmed through the test group. To enhance this analysis, we developed a novel nomogram model that integrates data from TCGA and GSE53624 to assess the 1-, 3-, and 5-year OS rates of ESCA patients, factoring in clinicopathological characteristics and risk scores. The calibration curves demonstrate an excellent match. These results indicated that the nomogram can be used for prognostic prediction.

In this study, the established ARG signature model was found to be closely related to the survival outcomes of patients with ESCA. The ARG signature included 5 ARGs: MYBBP1A, PLAU, BUB3, HOTAIR, and EHMT2. These genes are strongly associated with cancer. MYBBP1A has been verified to be verified to be of mitosis, cellular senescence, epigenetic control, cell cycle regulation, metabolic plasticity, and desiccation.^[34]^ PLAU promotes the progression of many tumors. For instance, PLAU facilitates the advancement of ESCA by converting fibroblasts into inflammatory cancer-associated fibroblasts through the uPAR/Akt/NF-κB/IL8 signaling pathway. PLAU serves as both a biomarker and a prognostic marker for breast cancer, contributing to resistance against trastuzumab.^[35,36]^ BUB3 belongs to the Bub protein family and plays a crucial role in the spindle assembly checkpoint. Silva et al noted levels were elevated in oral squamous cell carcinoma patients compared to normal cells, and suppressing Bub3 or Spindly proved toxic to these cancer cells while also enhancing their sensitivity to cisplatin treatment.^[37]^ Kang et al^[38]^ evaluated the effect of BUB3 on survival outcomes of surgically resected nonsmall cell lung cancer patients and found that Bub3 mRNA was highly expressed in tumor tissues, and the overall survival of patients with BUB3 rs7897156C > T was worse. In addition, BUB3 is also abnormally expressed in many human malignancy, such as prostate cancer, glioma tumors, colorectal cancer, breast cancer, hepatocellular carcinoma, adrenocortical carcinoma, and gastric cancer.^[39]^ HOTAIR is a prevalent long noncoding RNA in human malignancies influencing 17 different tumor types such as breast, cervical, esophageal squamous cell carcinoma, and colorectal cancers. Elevated levels of its expression enhance tumor spread, invasion, cellular proliferation, and movement, resulting in a grim prognosis and decreased overall survival.^[40]^ Kim et al^[41]^ showed that EHMT2 was significantly overexpressed in breast cancer tissues and that EHMT2 knockout reduced tumor cell migration/invasion. Fan et al revealed that individuals with prostate cancer exhibiting elevated EHMT2 levels possess a more advanced pathological grade and lower OS. EHMT2 is highly expressed in prostate cancer cell lines. EHMT2 knockout inhibits the proliferation of prostate cancer cells and induces apoptosis in prostate cancer cells.^[42]^

TME is the cellular environment in which cancer stem cells survive. Cancer stem cells can self-renew, and drive tumorigenesis. The TME consists of immune cells, lymphocytes, the extracellular matrix, fibroblasts, inflammatory cells from bone marrow, and signaling molecules.^[43]^ Our study found that individuals with greater risk scores exhibited increased ImuneScores, StromalScores, and ESTIMATEScores. The cell-type identification by estimating relative subsets of RNA transcripts analysis identified notable variations in the quantities of resting mast cells and Macrophages M2 between patients in the low- and high-risk categories. The ssGSEA analysis revealed that the statistically significant elements included CD56bright natural killer cells,” “CD56dim natural killer cells,” “MDSC,” “macrophages,” “monocytes,” “natural killer T cells,” “Regulatory T cells,” follicular helper cells,” “Type 1 T helper cells,” and Type 17 T helper cells.” Our research findings suggest that abnormal immune cell infiltration could be linked to the advancement of ESCA in patients.

We further analyzed whether the ARS could provide valuable insights for chemotherapy and targeted therapy. The IC50 value measures a drug’s ability to induce tumor cell apoptosis, a lower IC50 value indicates stronger induction capability. In our analysis, the IC50 values of 19 drugs were positively correlated with patient risk scores, suggesting that patients with ESCA with high ARS scores may experience greater drug resistance and might not benefit from treatment.

While our findings indicate that the ARS is effective in forecasting the outcomes for ESCA patients, it does have some drawbacks. Every ESCA case in this research was obtained from publicly accessible databases. Further external validation using clinical samples is required. Upcoming studies ought to delve deeper into the fundamental processes of ARS genes through experimental analysis.

## 5. Conclusion

In conclusion, our model predicted the survival outcomes among patients with ESCA based on ARGs. The immune condition and response to immunotherapy in ESCA patients were assessed as well. Combining anoikis with ESCA prognosis offers a new perspective for tumor-related research. Moreover, we analyzed the TME characteristics and drug sensitivity of patients with ESCA to better understand individualized treatment approaches.

## Acknowledgments

This study was supported by the Scientific Research Fund of the Hebei Provincial Health and Family Planning Commission (20220415). The author admits that the data of ESCA comes from the the Cancer Genome Atlas database and thanks to it. There is no conflict of interest in this article.

## Author contributions

**Conceptualization:** Yunwei Liang.

**Data curation:** Yunwei Liang, Xin Yin.

**Funding acquisition:** Yunwei Liang.

**Methodology:** Yunwei Liang, Xin Yin, Yinhui Yao, Ying Wang.

**Project administration:** Yunwei Liang, Ying Wang.

**Writing – original draft:** Yunwei Liang.

**Writing – review and editing:** Yunwei Liang, Ying Wang.

**Formal analysis:** Xin Yin.

**Visualization:** Yinhui Yao.

**Supervision:** Ying Wang.

## Supplementary Material


